# How Long Depends on How Fast—Perceived Flicker Dilates Subjective Duration

**DOI:** 10.1371/journal.pone.0076074

**Published:** 2013-10-23

**Authors:** Sophie K. Herbst, Amir Homayoun Javadi, Elke van der Meer, Niko A. Busch

**Affiliations:** 1 Berlin School of Mind and Brain, Berlin, Germany; 2 Humboldt-Universität zu Berlin, Berlin, Germany; 3 Institute of Behavioral Neuroscience, Department of Cognitive, Perceptual and Brain Sciences, University College, London, United Kingdom; 4 Institut für Medizinische Psychologie, Charité-Universitätsmedizin Berlin, Berlin, Germany; Duke University, United States of America

## Abstract

How do humans perceive the passage of time and the duration of events without a dedicated sensory system for timing? Previous studies have demonstrated that when a stimulus changes over time, its duration is subjectively dilated, indicating that duration judgments are based on the number of changes within an interval. In this study, we tested predictions derived from three different accounts describing the relation between a changing stimulus and its subjective duration as either based on (1) the objective rate of changes of the stimulus, (2) the perceived saliency of the changes, or (3) the neural energy expended in processing the stimulus. We used visual stimuli flickering at different frequencies (4–166 Hz) to study how the number of changes affects subjective duration. To this end, we assessed the subjective duration of these stimuli and measured participants' behavioral flicker fusion threshold (the highest frequency perceived as flicker), as well as their threshold for a frequency-specific neural response to the flicker using EEG. We found that only consciously perceived flicker dilated perceived duration, such that a 2 s long stimulus flickering at 4 Hz was perceived as lasting as long as a 2.7 s steady stimulus. This effect was most pronounced at the slowest flicker frequencies, at which participants reported the most consistent flicker perception. Flicker frequencies higher than the flicker fusion threshold did not affect perceived duration at all, even if they evoked a significant frequency-specific neural response. In sum, our findings indicate that time perception in the peri-second range is driven by the subjective saliency of the stimulus' temporal features rather than the objective rate of stimulus changes or the neural response to the changes.

## Introduction

The term “time perception” describes a variety of processes including the perception of durations from milliseconds to years, or judgments of temporal order and synchronicity, which have been discussed intensively in philosophy, psychology and cognitive neuroscience [1], [2]. In this study, we focus on perceived duration in the peri-second range—an important and prevalent subjective experience, whose underlying mechanisms still remain a puzzle [3]. We know that perceived duration of events does not always concur with objective time, as expressed by proverbs like “a watched pot never boils”. One reason for this is that time cannot be perceived by a dedicated sensory organ, and therefore perceived duration strongly depends on the content of the time interval (in addition to the interval's physical duration).

The idea that the content of a time interval affects its perceived duration can be found in the writing of the French philosopher Jean-Marie Guyau (1854–1888), who argues that “temporal experience is constructed based on the intensity of the stimuli, the number of stimuli, the attention paid to the stimuli, the associations of the stimuli, the extend of the differences between the stimuli and the expectations called by the stimuli.” [4] (cited from [5], pp. 31, 32). The relation between stimulus features and perceived duration of the stimulus has been central to psychological theories of time perception (see for instance [6]–[9]), and has set the ground for a host of scientific studies. Many of these studies refer to an influential proposal by Paul Fraisse, who suggested that the number of changes perceived during a time interval is of particular importance to the interval's perceived duration [10], p. 233. “Change”, in this context, can refer to any transformation of the stimulus across time, including changes in intensity, number, or space.

An important question is whether the effect of stimulus change on perceived duration is determined only by its objective frequency (the number of changes), or by the conscious perception, or by the neural processing of the change. For example, imagine a stimulus changing so rapidly that the change is not perceived as such. Would this stimulus' perceived duration be affected very much by the high frequency or very little because the change is not perceived? Answering this question will help to explore the mechanisms underlying duration perception [11].

**Figure 1:**
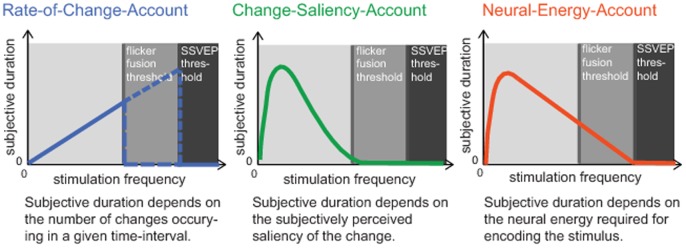
Three accounts describing the influence of objective flicker frequency, subjective flicker perception, and the neural response to flicker on subjective duration. We measured participants' flicker fusion threshold (the highest frequency perceived as flicker) and their SSVEP threshold (the highest frequency still evoking a significant frequency-specific SSVEP), and tested predictions from three accounts regarding how subjective duration is affected by flicker frequencies below, between and above these two thresholds. **Left panel:** The rate-of-change-account predicts that subjective duration increases monotonically with the objective rate of change (the flicker frequency) up to the flicker fusion threshold or the SSVEP threshold. **Middle panel:** The change-saliency account predicts that the effect of flicker frequency on perceived duration is maximal when the flicker is subjectively perceived as most salient. This should result in an inverted u-shaped relationship between flicker frequency and perceived duration, with a maximal effect at frequencies between 8–15 Hz. Invisible flicker (faster than the flicker fusion threshold) should not affect subjective duration, even if it evokes a frequency-specific neural response. **Right panel:** The neural-energy-account predicts that subjective duration depends on the neural energy expended in processing a stimulus. Hence, subjective duration should be longest for frequencies that evoke the largest neural responses (typically at 12–15 Hz). Note that in contrast to the change-saliency account, frequencies above the flicker-fusion threshold that still evoke a neural response should affect perceived duration.

Several accounts have been proposed that describe how perceived duration in the peri-second range could interact with the frequency of changes, their subjective perception, and their neural processing. We will briefly review the three most relevant accounts (see also Figure 1) and the predictions they make about the relationship between flicker frequency and perceived duration. In the remainder of this paper, we will focus specifically on visual flicker as one example of stimulus change.

### The rate-of-change-account

Fraisse has proposed that perceived duration is a function of the rate of perceived changes. A literal interpretation of this statement, to which we will refer as the “rate-of-change-account”, predicts a monotonous, possibly linear, increase of perceived duration with the stimulus' objective rate of change or frequency. Numerous studies have confirmed this account, showing that the more changes a stimulus undergoes, the longer it appears [12]–[18]. However, it is reasonable to assume that there is a limit for this effect such that changes which happen too fast to be perceived as changes cannot affect perceived duration. In fact, Fraisse [10] emphasized that the number of *perceived* changes determines perceived duration. In line with this assumption, we recently demonstrated that stimuli perceived as targets dilate the perceived duration of an interval, while stimuli that escape target-detection due to an attentional blink do not [19].

However, with reference to visual flicker processing, two limits need to be distinguished. First, a perceptual limit is defined by the flicker fusion threshold, beyond which a flickering stimulus is perceived as steady light [20]. It is important to note that stimuli flickering only slightly faster than the flicker fusion threshold still elicit a specific neural response to the flicker in spite of not being consciously perceived as flickering [21]–[24]. There is thus a second limit at the neural level — a frequency beyond which visual neurons do not respond to the flicker any more [25]. In sum, the “rate-of-change-account” predicts that the perceived duration of a flickering stimulus extends monotonically with increasing flicker frequency up to the perceptual, or neural threshold for flicker perception (see Figure 1, left panel).

### The change-saliency-account

Alternatively, perceived duration might be affected more by the subjective saliency of the changes, rather than by the objective rate of stimulus change. It has long been known that human observers are most sensitive to flicker at frequencies between 8–15 Hz [26], [27], while at higher frequencies, sensitivity decreases even well before the flicker fusion limit. In other words, flicker appears stronger or more salient at frequencies from 8–15 Hz.

Thus a “change-saliency-account” predicts an inverted u-shaped relationship between flicker frequency and perceived duration with a maximal effect at flicker frequencies between 8–15 Hz. In line with this proposal, Kanai et al. [15] found that the effect of flicker frequency on perceived duration saturates and reaches a plateau at 8–10 Hz, possibly because flicker saliency starts to wear off at this frequency range. Unfortunately, this study did not test frequencies beyond 12 Hz. Neither did a recent study by Plomp et al. [18], who showed temporal dilation induced by a 7 Hz modulation of the stimulus. Thus, it is currently unknown whether the effect of flicker on perceived duration remains at a stable plateau or decreases at frequencies faster than 12 Hz, as predicted by the “change-saliency-account” (see Figure 1, middle panel). However, this account clearly predicts no influence of flicker frequency on perceived duration for frequencies above the flicker fusion threshold.

### The neural-energy-account

Recently, Eagleman and Pariyadath [9] have proposed that the more energy is expended in representing a stimulus, the longer its perceived duration. This hypothesis is based on the observation that unpredictable stimuli are perceived as longer, and at the same time evoke a stronger neural response. For example, rare oddball stimuli appear subjectively prolonged in duration [28], [29] while repeated standard stimuli appear shorter, and the amplitude of the cortical response diminishes with stimulus repetition [30]. The “neural-energy-account” can explain effects of stimulus magnitude on perceived duration, such as prolonged duration for filled versus empty intervals [31]. While Eagleman and Pariyadath did not define how exactly “neural energy” is related to a neural response (i.e. to spike rate, membrane depolarization, etc.) [9], p. 1846, they suggested that perceived duration is mostly influenced by early sensory processing. Along the same line, Kanai et al. suggested that “the source of temporal cues resides in early visual areas” [15], p. 1429. The neural response to visual flicker can be observed in the EEG as the steady state visual evoked potential (SSVEP): evoked oscillations measured over early visual cortex at the same frequency as the entraining stimulus [22], [32]–[34]. Therefore, SSVEP reflect a specific neural response to the flicker, and their amplitudes can be measured as a signature of the neural energy involved in coding a flickering stimulus. Thus, for each flicker frequency, stimulus duration may be perceived as longest by those observers whose SSVEP amplitude is particularly large at that frequency. Moreover, SSVEP amplitudes show a peak at frequencies of around 12–15 Hz and steadily decrease at higher frequencies [34]. Thus, the neural-energy-account also predicts that the effect of flicker on perceived duration peaks around 12–15 Hz and then decreases at higher frequencies (see Figure 1, right panel). Note that this prediction parallels the change-saliency-account, but contradicts the rate-of-change account, which predicts that the effect of flicker on perceived duration *increases* with flicker frequency. However, in contrast to the change-saliency-account, the neural-energy account predicts an influence of flicker on perceived duration even for frequencies above the flicker fusion threshold as long as they evoke an SSVEP.

### The present study

In the present study, we tested predictions derived from three accounts describing the influence of flicker frequency on perceived duration (see above). Specifically, the rate-of-change account predicts that perceived duration increases steadily with increasing frequency up to the perceptual or neural flicker threshold. By contrast, the change-saliency account predicts an inverted u-shaped relationship between perceived duration and flicker frequency, peaking at the point where the flicker subjectively appears most salient. According to this account, there should be no effect above the flicker fusion threshold. Finally, the neural-energy account predicts that perceived duration is correlated with the amplitude of the neural response to flicker and decays only around the neural flicker threshold.

To validate these predictions, we presented flickering stimuli of different duration and frequency, including frequencies around the perceptual and neural flicker threshold. We assessed each observer's individual flicker fusion threshold (the highest flicker frequency perceived as flickering) and their SSVEP threshold (the highest flicker frequency that evoked an SSVEP). Finally, we estimated the stimuli's perceived duration with respect to these two thresholds.

We found that perceived duration is strongly dilated by slow flicker frequencies, and that this effect wears off around the perceptual flicker fusion threshold. Although we found measurable SSVEP for frequencies higher than the flicker fusion threshold, flicker at these frequencies had no effect on perceived duration. Thus, in sum, we argue that our results support the change-saliency-account.

## Methods

### Participants

30 participants (29 students and the first author; 6 male; mean age: 24.4 years, *SD*: 6.1 years; 6 left-handed) took part in the experiment after giving written informed consent. Students received course credits in return for participation. The study was carried out in accordance with the Declaration of Helsinki. Ethics approval was obtained from the ethics committee of the Department of Psychology at Humboldt-University, Berlin (reference number 2011-06). No participant reported a history of epilepsy or neurological illness.

### Stimuli and apparatus

Stimulus presentation and the collection of behavioral responses were achieved using the psychophysics toolbox V3 [35], [36] for MATLAB. Responses were entered on a conventional computer keyboard using index and middle fingers of the right hand.

Flickering light was presented using a pair of custom built flicker goggles. Two light-emitting diodes (LEDs) were attached to conventional swimming goggles pointing towards participants' eyes and covered by matte paper. Both eyes were stimulated simultaneously with white light with 43 cd/*m* ^2^ maximum luminance. Stimulation on both eyes was controlled by a computer with a maximum operating speed of 500 Hz (1 ms on and 1 ms off).

The frequencies used for stimulation were chosen according to the features of the goggles (by specifying the time the LEDs were switched on and off). The on- and off-periods were of equal duration, such that total light exposure across the whole stimulation period was kept constant across all frequencies. We used the following on- and off- periods: 3, 5.5, 6, 7, 8, 9, 12, 16, 32, 64 ms, resulting in frequencies of 7.7, 15.7, 31.1, 41.7, 55.4, 62.3, 71.1, 82.9, 90.6, and 165.7 Hz, due to a tolerance of +0.015 ms on each switching. During the third phase of the experiment, we used a slightly different range of frequencies, to also cover lower frequencies. Each stimulation phase started and ended with half an on-period, to make sure the stimulus was perceivable for the time specified (and not shorter, which would have been the case if the last cycle ended with an off-period).

### EEG recording

EEG was recorded with a BioSemi Active-Two amplifier system from 64 active Ag/AgCl electrodes arranged according to the extended 10–20 international system for electrode placement [37]. To monitor for eye movements and blinks, the horizontal and vertical electro-oculogram (EOG) was recorded. Furthermore, signals were recorded from two electrodes at the left and right mastoid for later referencing. Two additional electrodes (CMS: Common Mode Sense and DRL: Driven Right Leg) were used as reference and ground. Signals were sampled at 2048 Hz with 24 bit resolution and filtered between 0.16 Hz and 215 Hz.

Data were analyzed offline with the EEGlab V10 toolbox for Matlab, *sccn.ucsd.edu/wiki/EEGLAB*, [38]. Data were downsampled to 512 Hz, high-pass filtered at 1 Hz, and re-referenced to the average of the two mastoids. We applied a notch filter between 49 and 51 Hz to eliminate ambient electrical noise. The continuous data were epoched into intervals of −0.5 s to 30 s around stimulus onset.

Artifacts were rejected in two steps. First, trials containing non-stereotypical artifacts were removed manually based on visual inspection (overall 8.2% of trials). In a second step, independent component analysis (ICA) was applied to the remaining trials (and only the clean channels). ICA components reflecting eye blinks or noise were identified by visual inspection and removed. Noisy channels were replaced by interpolating neighboring channels after the ICA procedure.

### Paradigm, procedure and analysis

The experiment was performed in three phases, two of which were measured on the first day, and the third on another day within one week. Participants were seated comfortably on a chair in a dark and electrically shielded testing chamber. In the first phase, we recorded EEG and analyzed the steady state visual evoked potential (SSVEP) as a measure of the neural response to flicker. In the second phase, flicker fusion thresholds were acquired. In the third phase, we measured the perceived duration of the flickering stimuli. All data were analyzed using MATLAB (R2010; The MathWorks Inc.) and SPSS (19, SPSS Inc., IBM Statistics 2010).

#### Phase I: Neural response evoked by flicker

Participants were presented with 30 s of flickering light on each trial. Pilot studies had shown that 30 s of stimulation reliably evoke measurable SSVEP, even at high frequencies. On each trial, participants reported whether they had seen flicker or steady light. Note that in phase I, subjective perception of flicker was assessed only to assure that participants remained alert, while flicker fusion thresholds were actually measured in phase II. We presented five trials of 30 s at each frequency (50 trials in total). Frequencies were randomly intermixed and presented in four blocks. Between blocks participants could take breaks of self-determined length. Participants were asked to keep their eyes steady and avoid closing their eyes and blinking during stimulus presentations.

SSVEP were analyzed by computing a Fast Fourier Transform (FFT) of the average of the five trials for each flicker frequency. The first two seconds of each epoch were not included in this analysis in order to omit the onset-evoked ERP. Furthermore, following suggestions by Bach et al. [39], the length of the data on which the FFT was computed for each flicker frequency was adjusted to yield integer multiples of the duration of the flicker cycle. The resulting amplitude spectra were averaged across all channels. SSVEP amplitudes were quantified as the FFT amplitude at the stimulation frequency.

We tested whether a significant SSVEP was evoked at a given stimulation frequency using a bootstrapping procedure. For each participant, we compared the amplitude spectrum evoked by a given stimulation frequency to a resampled comparison spectrum. Five random samples were drawn with replacement from the single trials of all conditions, the FFT spectrum was computed on their average, and this procedure was repeated 5000 times. The SSVEP at a given frequency was considered significant if the corresponding peak in the FFT spectrum was larger than 0.1% of the peaks in the resampled spectra (p<0.001).

#### Phase II: Flicker fusion thresholds

The same flickering stimuli were presented as described above, but of 2 s duration—the same stimulus duration as used in phase III for the assessment of perceived duration. 20 trials per stimulation frequency were presented in four blocks, separated by self-paced breaks. On each trial, participants indicated with a button press whether they had perceived a stimulus as flickering or as steady light.

In order to calculate the individual flicker fusion threshold for each stimulation frequency, we fitted logistic sigmoid curves to the proportions of yes/no responses, using the psignifit toolbox for MATLAB, psignifit.sourceforge.net, [40]. Bayesian inference was used to estimate the parameters of the psychometric function. The 90% threshold of the psychometric function (i.e. the stimulus was reported as “steady” on 90% of trials) was used as an estimate for the flicker fusion threshold.

10% is commonly assumed as the lapse rate in psychophysical experiments [40]. Thus, using a cut-off as high as 90% allowed us to make sure that frequencies classified as “not perceived as flickering” were in fact perceived as steady. This was particularly important for defining the frequency range in which flicker was perceived as steady, but still evoked SSVEP (see below).

#### Phase III: Perceived duration

Perceived duration of the flickering stimuli was assessed in a two-alternative-forced choice task. Participants were presented with two stimuli on each trial: one standard stimulus (2 s long) flickering at a reference frequency of 165.7 Hz and one test stimulus of variable duration (0.5–3.5 s) at one of eleven frequencies (3.9–165.7 Hz). Note that flicker at 165.7 Hz was perceived as static by all observers. The order of standard and test stimuli was counterbalanced across the trials. We used a quasi-static reference frequency of 165.7 Hz rather than a non-flickering reference stimulus for two reasons. First, this frequency was expected to be always perceived as static. Second, and more importantly, a non-flickering stimulus would have had a 100% on-period, and therefore been brighter than any flickering stimulus with 50% on/50% off-periods. Participants indicated with a button press which of the two stimuli lasted longer. After each response, a brief tone indicated that the response had been registered. 40 trials were presented for each frequency, divided into 20 shorter and 20 longer trials (440 trials total). The testing was performed in four blocks, separated by self-paced breaks.

Our study design differs from frequency adaptation studies [41]–[44]. These studies used sub-second durations to show that adaptation to 20 Hz flicker leads to subsequent duration compression of stimuli flickering at 5 or 10 Hz. Importantly, flicker adaptation does not seem to require conscious perception of the flicker [45] indicating a different underlying mechanism.

We used an adaptive staircase procedure (Quest, [46]) to estimate each participant's subjective duration separately for each stimulation frequency. Two independent staircases were interleaved for each frequency for presenting test stimuli longer and shorter than the 2 s standard. The staircase procedure estimated the duration of test stimuli that was required for 82% correct classification as longer or shorter than the standard. An 82% performance criterion is recommended for two-alternative-forced choice tasks by the authors of the Quest procedure [46] because it results in the most reliable estimates.

For a stimulus that is neither over- nor underestimated, both staircases should estimate the same absolute difference to the standard duration. For instance, a value of 0.2 s for longer test stimuli and 0.2 s for shorter test stimuli would indicate that the test stimulus needs to last 2.2 s to be perceived with 82% accuracy as longer, or 1.8 s to be perceived as shorter, respectively. Thus, we quantified the over- or underestimation of stimulus duration by subtracting the respective differences from the standard duration (2 s) for long from those for short stimuli (in the above example this would result in 0.2−0.2 = 0 s). Estimates that differed from the standard duration by more than 2 s were considered outliers (as in the case of shorter durations, this would have resulted in negative duration values) and removed from further analyses.

To test whether flicker frequency influences perceived duration, we computed a repeated measures ANOVA with the factor “frequency” (11 levels). To assess at which frequency the estimated subjective duration indicated a significant overestimation of duration, post hoc one-tailed paired-samples *t*-tests were subsequently performed for each frequency separately, comparing the estimated subjective duration for each frequency against the estimated duration for the quasi-static reference frequency (165.7 Hz). *P*-values were corrected for multiple comparisons by using the Bonferroni-Holm procedure [47].

Note that a non-significant *t*-test does not, strictly speaking, provide evidence for the null hypothesis, which is that perceived duration was unaffected by the flicker of a frequency. Thus, we complemented this analysis with the calculation of Bayes factors ([48]; using the online calculation tool provided at *pcl.missouri.edu/bf-one-sample*), which can indicate not only evidence for the presence of an effect, but also for its absence. Bayes factors indicate the ratio between the conditional probabilities of the null and alternative hypotheses [49], [50]. As suggested by Rouder et al. [51], Bayes factors were computed using uninformed priors, in order not to bias the test result.

## Results

The experiment consisted of three phases, in which we independently assessed SSVEP thresholds, flicker fusion thresholds, and the subjective duration of flickering stimuli (see Methods for a detailed description of the three different measures). In all three phases, we used custom-designed flicker goggles to present flickering light (ranging from 3.9 Hz to 165.7 Hz) to both eyes simultaneously.

We will first present the results of these three phases separately before integrating all results to test predictions made by the three accounts of how stimulus change is related to perceived duration.

### Determination of thresholds and subjective duration

#### Phase I: Neural response evoked by flicker (SSVEP thresholds)

In Phase I, we presented flickering light at different frequencies (7.7–165.7 Hz) with a constant duration of 30 s. For each stimulation frequency, steady state visual evoked potentials (SSVEP) were quantified as the amplitude of the power spectrum of the EEG signal at that frequency. Thus, SSVEP represent a specific neural response to a stimulation frequency. SSVEP thresholds—the highest frequency evoking a significant SSVEP amplitude peak according to the bootstrap procedure—varied considerably across participants, from 15.7 Hz to 165.7 Hz (the latter was found in six participants) with an average of 87.0 Hz (*SD* = 49.6 Hz).

While no study has previously addressed the upper frequency limit for observing SSVEPs, we found one report on brain responses to frequencies above the individual flicker fusion threshold [51]. In this study, some participants did not show an SSVEP at high frequencies, confirming the considerably high variation in individual SSVEP thresholds.

**Figure 2:**
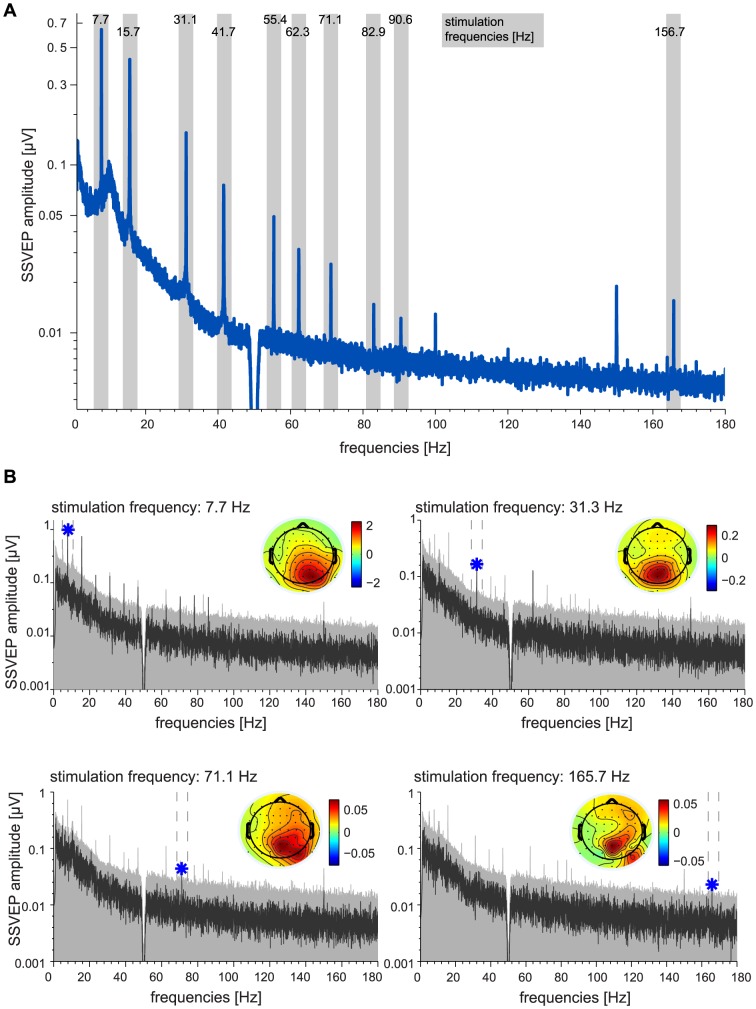
Steady State Visual Evoked Potentials. **A:** Grand-average frequency spectra for 30 participants as recorded during phase I, based on 30 s stimulation intervals and averaged across all channels. Gray shades indicate a ±2 Hz range around stimulation frequencies (7.7 Hz to 165.7 Hz). Note that spectral peaks indicating steady state evoked potentials were found even at the highest stimulation frequencies that were never perceived as flickering. **B:** Data of an exemplary participant. Frequency spectra evoked by four stimulation frequencies: 7.7 Hz, 31.3 Hz, 71.1 Hz, 165.7 Hz (dark gray). The light gray area indicates the 99.9%-percentile of the resampled data, as used to determine statistical significance of peaks at the stimulation frequency. Blue stars indicate significant amplitude peaks at the stimulation frequency (p<0.001). The topographies show the scalp distribution of amplitudes at the peak frequency.

SSVEP amplitudes averaged across all participants are shown in Figure 2A. Figure 2B shows SSVEP amplitudes and topographies from an exemplary participant.

#### Phase II: Flicker fusion thresholds

**Figure 3:**
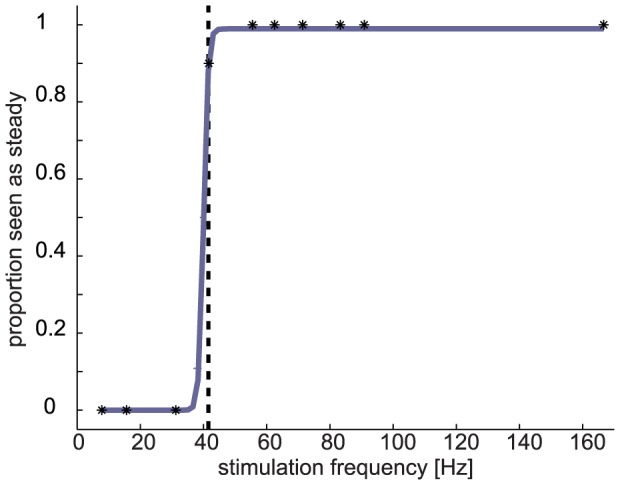
Subjective Flicker Perception. Psychometric function as determined in phase II, describing the relation between stimulation frequency and flicker perception (for the same participant as in Figure 2). The dashed vertical line shows the 90% threshold of the curve, at which the stimulus was reported to be “steady” in 90% of trials. This frequency was taken as the flicker fusion threshold.

**Figure 4:**
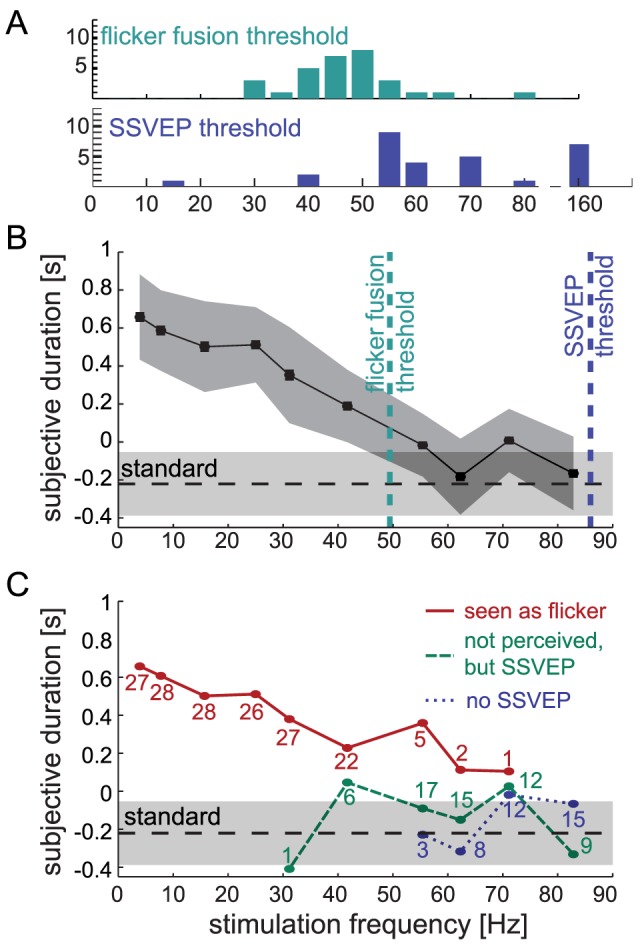
Perceived Duration. **A:** Histogram of individual flicker fusion thresholds (green) and SSVEP thresholds (blue) as determined in phases I and II of the experiment. **B:** Average perceived duration for all stimulation frequencies (positive values on the y-axis indicate over-estimation, negative values indicate under-estimation). The dark gray area indicates 95% confidence intervals. The dashed horizontal line shows the threshold estimated for the reference frequency (165.7 Hz) against which all other frequencies were compared (the light gray shade indicates 95% confidence interval). Frequencies up to 41.7 Hz were significantly perceived as longer than the standard. Vertical lines show the flicker fusion threshold (cyan) and the SSVEP threshold (blue) averaged across participants. **C:** Same data as in B, shown separately for stimuli perceived as flicker, not perceived as flicker but evoking an SSVEP, and no SSVEP. The duration of stimuli perceived as flicker (red) was overestimated compared to the reference frequency, for frequencies up to 31.4 Hz (all p<0.05), resulting in a linear relationship between flicker frequency and perceived duration. Flicker frequencies that were not perceived as flicker but evoked a frequency-specific SSVEP (green), and frequencies that were neither perceived nor evoked a SSVEP (blue) were not perceived as longer than the reference frequency. The numbers next to the data points indicate the number of participants contributing to this data point (if they do not add up to 30, this is because of excluded outliers).

In Phase II, we presented flickering light at different frequencies (7.7–165.7 Hz) with a constant duration of 2 s. Flicker fusion thresholds were estimated in a yes/no-task, computed for each individual as the 90% threshold of the psychometric function (the stimulus being rated as steady in 90% of trials). Thresholds ranged from 30.6 Hz to 81.9 Hz (*mean* = 49.4 Hz, *SD* = 10.3 Hz). These rather high flicker fusion thresholds were probably due to the strong stimulus luminance and the illuminated area (i.e. the entire visual field) [52]. Figure 3 shows the psychometric curve fitted to the data from an exemplary participant. As expected, flicker fusion thresholds were significantly lower than SSVEP thresholds (the highest frequency at which a significant SSVEP was evoked; one-tailed paired-samples *t*-test, T(29) = 3.93; p<0.001). Thus, there was a range of flicker frequencies that evoked a frequency-specific neural response even though they were not perceived as flickering. Howver, flicker fusion thresholds and SSVEP thresholds did not significantly correlate across participants. The distribution of flicker fusion thresholds for all participants is shown in Figure 4A.

#### Phase III: Subjective duration

In Phase III, we presented on each trial a standard with constant duration (2 s) at the reference frequency (165.7 Hz), and a test stimulus with variable duration and frequency (0.5–3.5 s, 3.9–165.7 Hz). The order of standard and test stimuli was counterbalanced. Subjective duration of the flickering stimuli was estimated by two interleaved staircases, quantifying the duration a given stimulus needed to have in order to be classified as longer/shorter than the standard with 82% accuracy (for a detailed description of the procedure, see Methods, p. 6). We then subtracted the estimates for under-estimation from estimate for over-estimation to obtain a single measure for subjective duration (see Table 1).

**Table 1:** Subjective duration (i.e. difference from the 2 s standard stimulus) estimated by the Quest algorithm for each stimulation frequency (mean of all 30 participants).

frequency (Hz)	mean duration estimates (s)	adjusted p-value	Bayes factor
3.91	0.66	<0.001	0.002
7.71	0.59	<0.001	0.002
15.71	0.50	<0.001	0.01
25.00	0.51	<0.001	0.001
31.14	0.35	= 0.01	0.12
41.71	0.19	= 0.04	0.38
55.43	−0.02	= 0.07	0.74
62.29	−0.18	= 0.88	7.06
71.14	0.01	= 0.35	3.47
82.86	−0.17	= 0.88	6.99
165.7	−0.22	used as reference	

One-tailed paired-samples *t*-tests (corrected for multiple comparisons with the Bonferroni-Holm method) indicate that for frequencies up to 41.7 Hz the estimated duration significantly differed from the duration estimated for the reference frequency; stimuli flickering at these frequencies were perceived as longer. The last column shows Bayes factors, corresponding to the ratio between the probabilities of the null and alternative hypotheses. Bayes factors <0.3 can be taken as evidence for an effect of flicker frequency on perceived duration, while Bayes factors ≥3 indicate that there is likely no such effect. Intermediate values allow no conclusive decision for either of the two hypotheses.

Subjective duration was clearly affected by flicker frequency: the duration of slowly flickering stimuli was perceived as longer than the quasi-steady reference (165.7 Hz). For example, a 2 s stimulus flickering at 3.9 Hz was overestimated by about 0.66 s, meaning it had to be presented for only 1.34 s to be perceived as equal to the standard duration of 2 s (see Figure 4B). This effect was confirmed by a repeated measures ANOVA with the factor stimulation frequency (11 levels; *F*(10,290) = 15.42; *p*<0.001, *η* ^2^ = 0.35). Within-subject contrasts revealed a strong linear effect (*F*(1,29) = 63.12; *p*<0.001; *η* ^2^ = 0.69; also significant but of smaller effect size was the cubic effect: *F*(1,29) = 5.24; *p* = 0.02; *η* ^2^ = 0.15). These results show that flicker frequency dilates perceived duration, and that the effect decreases linearly with increasing stimulation frequency.

To test at which frequencies stimuli were perceived as longer than the reference frequency (165.7 Hz), we computed post hoc one-tailed t-tests comparing the estimated subjective duration for each frequency against the estimated duration for the reference frequency. Table 1 shows the estimated subjective durations and *p*-values corrected for multiple comparisons with the Bonferroni-Holm method [47]. Subjective duration for frequencies up to 41.7 Hz was significantly longer than the duration of the reference frequency (corrected p-values<0.05). At 55.4 Hz, subjective duration was only marginally longer (p = 0.071).

While this result implies that for frequencies of 55.4 Hz and higher, the null hypothesis (i.e. no effect of flicker on perceived duration) cannot be rejected in favour of of the alternative hypothesis (i.e. flicker does affect perceived duration), the result of a failed *t*-test has no direct implications for actually *accepting* the null hypothesis, even when the alternative hypothesis is rejected based on a too large *p*-value. To assess which of the two hypotheses is more likely, we calculated Bayes factors for each frequency (see Methods and Table 1). Bayes factors allow comparing the probability of two alternative hypothesis directly, without specifying an arbitrary cut-off criterion (such as p = 0.05). According to Rouder et al. [48], a Bayes factor smaller than 0.3 provides “some evidence” for the alternative hypothesis (meaning, it is three times as likely given the data), while a Bayes factor larger than three provides “some evidence” for the null hypothesis. We confirmed an effect of flicker frequency on perceived duration for frequencies up to 31.2 Hz (all Bayes factors <0.3). At 41.7 Hz (Bayes factor of 0.37) an effect of flicker frequency on perceived duration was almost three times as probable as no such effect, confirming the result of the *t*-test. At 55.4 Hz (Bayes factor of 0.70) an effect of flicker frequency on perceived duration was about 1.3 times as probable as no effect, which provides inconclusive evidence for either hypothesis. At flicker frequencies above 55.4 Hz (all Bayes factors ≥3) all evidence suggests that flicker had no effect on perceived duration.

### The interplay between neural processing and conscious perception of flicker and perceived duration

The threshold frequencies found in phase I and II of the experiment (see Figure 4A for the distribution of threshold frequencies) allowed to define for each participant three frequency ranges of interest: (1) flicker frequencies that are perceived as flickering and evoke a frequency-specific neural response, (2) frequencies that are too fast to be perceived as flickering, but nonetheless evoke a frequency-specific neural response, and (3) even faster frequencies which are neither perceived as flickering nor evoke a frequency-specific neural response. The central aim of this study was to clarify the effect of flicker on subjective duration in each of these frequency ranges. To this end, we tested perceived duration separately for flicker frequencies in the three frequency ranges described above (see Figure 4C). Note that threshold frequencies varied across participants, which is why frequency ranges were defined individually for each participant (for the number of observations at each frequency, see Figure 4C).

The duration of flickering stimuli was significantly overestimated provided the stimuli were perceived as flickering (frequency range 1: 3.9 Hz–81.9 Hz). When each of these frequencies was tested separately against the reference frequency, frequencies up to 31.1 Hz produced significant overestimation of the stimulus' duration (all *p*<0.05, corrected for multiple comparisons). However, at 41.7 Hz, there was only a marginal overestimation-effect (*p* = 0.059, corrected), and no effect was found at 55.4 Hz, presumably due to the small number of observations (only 5 participants reported 55.4 Hz as flickering). Bayes factors of 0.46 at 41.7 Hz and 0.99 at 55.4 Hz indicated that despite an effect of flicker on perceived duration at these frequencies is more likely at least at 41.7 Hz, the data do not allow for a clear decision between the alternative hypothesis and the null hypothesis. Flicker frequencies that were not perceived as flickering, but nonetheless evoked SSVEP (frequency range 2: 31.1 Hz–165.7 Hz) had no effect on perceived duration (all corrected p-values ≥0.85; Bayes factors ≥2.08). Similarly, frequencies that were neither perceived as flickering, nor evoked an SSVEP (frequency range 3: 55.4 Hz–165.7 Hz) did not affect perceived duration (all corrected p-values ≥0.45; Bayes factors ≥1.07). In sum, flickering stimuli appeared longer than static stimuli only when they were perceived as flickering. This effect was maximal at lower frequencies, which were most consistently perceived as flickering. By contrast, no effect of flicker on perceived duration was found for frequencies above the flicker fusion threshold, even if these stimuli evoked a significant frequency-specific neural response to the flicker.

**Figure 5:**
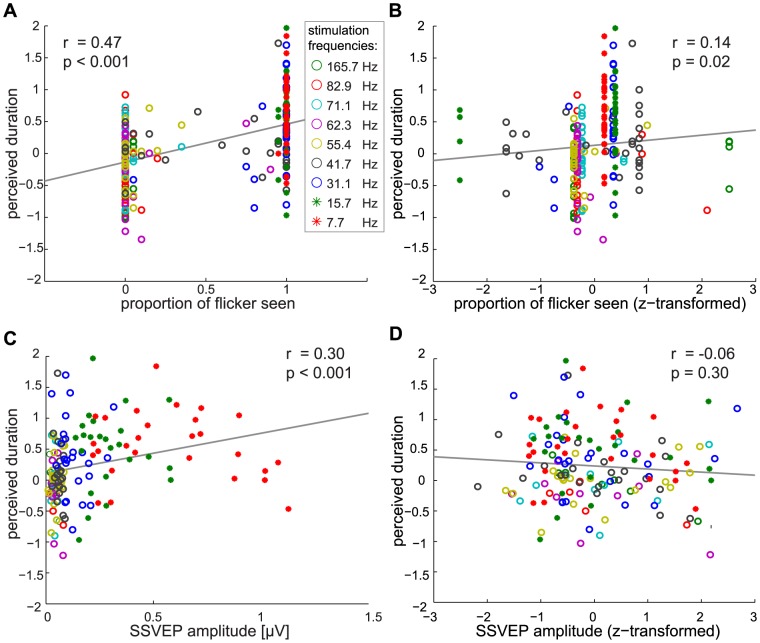
Correlation Analyses. **A:** Correlations between proportion of trials seen as flicker, and perceived duration. Although very high and low frequencies did not show great variation in perception of flicker, overall, conscious perception of flicker led to longer perceived duration. (*r* = 0.47; *p*<0.001). **B** After a z-transform, which removed all variance between frequencies while keeping the variance within frequencies, a significant correlation remained between flicker perception and perceived duration (*r* = 0.14; *p* = 0.02). This result indicates that participants who perceived a given frequency as flicker perceived stimulus duration as longer than those who did not perceive this frequency as flicker. **C:** SSVEP amplitudes evoked by each flicker frequency significantly correlated with perceived duration (*r* = 0.30; *p*<0.001). **D:** A z-transform of SSVEP amplitudes, which removes the between-frequency effect, removed the correlation between SSVEP and subjective duration (*r* = −0.06; *p* = 0.30), indicating that participants with stronger SSVEP at a given frequency did not perceive stimuli of that frequency as longer.

To complement the previous analysis, we directly analyzed the correlation between perceived duration and how often a frequency was perceived as flickering. Both factors were significantly correlated (*r* = 0.47, *p*<0.001; Figure 5A). Note that this correlation may have been influenced by two possible sources of variance: a between-frequency effect of objective flicker frequency (i.e. lower frequencies affected perceived duration more) and a within-frequency effect of subjective perception of flicker (i.e. perceiving a stimulus as flickering affected perceived duration, independent of frequency). Both factors are obviously related. To analyze the specific correlation between perceived duration and *subjective* flicker perception, we z-transformed the proportion of stimuli perceived as flickering using the mean and standard deviation of each frequency separately. Thus, the z-scored measures of flicker perception had the same mean and standard deviation at all frequencies. This amounts to removing all variance between flicker frequencies while keeping the within-frequency variance due to subjective flicker perception across participants. A significant correlation between the z-scored measures (*r* = 0.14; *p* = 0.02) confirmed that participants who subjectively perceived flicker for a frequency judged stimuli of that frequency as longer (Figure 5B). This positive relation between subjective flicker perception and perceived duration provides strong evidence for the change-saliency account: the stronger the subjective perception of the flicker, the longer its perceived duration.

Similarly, we analyzed the correlation between perceived duration and SSVEP amplitudes. Both factors were significantly correlated: the larger the SSVEP evoked by the flicker, the more the stimulus duration was overestimated (*r* = 0.30, *p*<0.001; Figure 5C). Again, this correlation could be due to two possible sources of variance: a between-frequency effect (i.e. lower frequencies evoke larger SSVEP) and a within-frequency effect of individual responsiveness to flicker (i.e. participants with larger SSVEP show stronger overestimation, independent of frequency). To remove the between-frequency effect on SSVEP amplitudes, we z-scored SSVEP amplitudes separately for each frequency, thus removing the between-frequency variance. The z-scored SSVEP amplitudes were not significantly correlated with perceived duration (*r* = −0.06, *p* = 0.30, see Figure 5D), indicating that participants with stronger neural responses did not judge the stimulus duration as longer.

## Discussion

How does the subjective duration of an event depend on the number of changes within the event? To answer this question, we presented fast-changing visual stimuli (i.e. flicker) and studied how their subjective duration depends on the flicker's objective frequency, the subjective perception of the stimulus as flickering, and the neural response to the flicker. Flicker frequencies up to 50 Hz were perceived as flickering by most participants. In the range from 50–87 Hz, flicker was not perceived as flickering even though a significant frequency-specific neural response to the flicker was observed in most participants. At frequencies above 90 Hz, flicker was never perceived and rarely evoked a significant frequency-specific response. These findings allow an evaluation of three accounts that describe the relationship between subjective stimulus duration and stimulus frequency.

The rate-of-change-account predicts a monotonous increase of perceived stimulus duration with flicker frequency (the rate of change of the stimulus) as long as the flicker is perceived as flickering, or evokes a frequency-specific neural response (see Figure 1A). This prediction is clearly not supported by the data. Only slowly-flickering stimuli caused strong overestimation of the stimuli's duration; a frequency of 4 Hz dilated perceived duration by about 30% compared to a steady control stimulus, confirming the notion that the rate of changes during a time interval affects its subjective duration [4], [10], [12]. However, this effect decreased with frequency: a frequency of 40 Hz (which was still consistently perceived as flicker) dilated perceived duration only by about 10% (Figure 4B). Thus, subjective stimulus duration cannot be explained by the objective number of events within an interval.

Our findings line up with a number of previously published results that challenge the rate-of-change-account and the assumption that stimulus change is linearly related to temporal accumulation. For example, Matthews [53] found that stimuli at constant speed are judged as longer compared to decelerating and accelerating stimuli, which cannot be explained by classical accumulation models. Similar results are reported by Binetti et al. [54], who compared temporal judgments for decelerating and accelerating flickering stimuli and found symmetrical over and underestimation compared to a constantly flickering comparison stimulus, which contradicts the rate-of-change-account, since all stimuli contained equal number of changes. Bruno et al. [55] found that drifting stimuli are judged as longer than static stimuli, which confirms the rate-of-change-account. However, they also find mixed stimuli (including drifts and static periods) to be judged longer than the drifting ones, which cannot be explained by the rate-of-change-account, but requires an additional explanatory variable, such as reduced predictabilty of the mixed stimulus. Thus, several studies have found evidence against a simple linear relationship between stimulus change and perceived duration. In sum, these findings suggest that perceived duration results from a more complex cognitive computation, including contextual variables and not just the physical properties of the stimulus itself.

The change-saliency-account assumes that perceived duration depends on the subjective saliency of the flicker, which is strongest in the range of 8–15 Hz [26], [27]. This account predicts an inverted u-shaped relationship between flicker frequency and perceived duration. Flicker at frequencies above the flicker fusion threshold should not influence perceived duration, even if it evokes a frequency-specific neural response (Figure 1B). In line with this proposal, the effect of overestimation was strongest at slow frequencies. The effect decreased linearly with increasing frequency and was absent for frequencies above the flicker fusion threshold (see Figure 4 B). Interestingly, the Bayes factors did not increase linearly with increasing frequency, but showed a jump from a value of 0.74 at 55.43 Hz to 7.06 at 62.29 Hz, indicating very low probability that flicker affects perceived duration beyond that frequency. Since this frequency range also represents the average flicker-fusion threshold, this finding further supports our conclusion that subjective perception of flicker, rather than the objective flicker frequency, is crucial for perceived duration. Furthermore, this finding may indicate that difference perceptual processes underlie the timing of subjectively changing and non-changing stimuli.

The strongest time dilation effect occurred at about 4 Hz, followed by a steady decrease of the effect. The lack of a ‘rising phase’ of the effect (as predicted in Figure 1B) could have resulted from the exclusion of frequencies lower than 4 Hz in the spectrum of frequencies tested. Also, the broad range of frequencies used may have shifted the subjective saliency of the flicker to be greatest for the stimuli that were clearly seen as flickering (the lowest frequencies), while most other stimuli appeared steady. Further support for the change-saliency-account comes from the result that even within a given flicker frequency, subjective duration was correlated with how often participants saw the stimulus as flickering (Figure 5A). In sum, the effect of flicker on subjective duration was modulated by the flicker's saliency and was dependent on its conscious perception as flickering.

The neural-energy-account assumes that subjective duration is directly related to the neural energy expended in representing the stimulus [9]. Due to the inverted u-shaped relation between flicker frequency and SSVEP amplitude [34], this account predicts a u-shaped relationship between flicker frequency and perceived duration, similar to the change-saliency-account (Figure 1C). As described above, the results are in line with this prediction. However, the neural-energy-account additionally predicts that a flickering stimulus that evokes a frequency-specific neural response should influence subjective duration, even if it is not perceived as flickering. Moreover, the strength of a participant's neural response to a given flicker frequency should be correlated to the participant's subjective duration of stimuli of that frequency. Neither of these predictions was supported by our results. First, frequencies that were not perceived as flickering but nonetheless evoked a significant SSVEP had no effect on subjective duration (Figure 4). Second, the strength of a participant's SSVEP for a given frequency did not correlate with the subjective duration of stimuli at that frequency. One reason for this lack of correlation could be the high variability of the SSVEP thresholds across participants. It is important to keep in mind, that not measuring a significant SSVEP amplitude at a given frequency for a particular participant means that our measurement and bootstrap procedure could not identify a significant amplitude at this frequency, but not necessarily that there was no response evoked by the stimulus. Furthermore it should be noted that the original notion of the neural-energy-account [9] does not specify any particular neural process. Thus, it is conceivable that the SSVEP, despite being a neural correlate of flicker processing, does not reflect the critical properties of “neural energy”, and therefore does not relate to the effect of flicker on subjective duration. However, the results do seem to rule out that subjective stimulus duration is grounded in the low-level neural processing of the visual stimuli.

### Conclusion

In sum, temporal frequency asserts a strong influence on subjective duration, but only when the frequency is consciously perceived as flickering. The effect is strongest for slow frequencies which evoke the most salient percept of change, therefore supporting the assumption that the subjective saliency of the change determines perceived duration. SSVEP amplitudes, measured as a signature of neural coding energy, are not related directly to perceived duration. Thus, our findings argue against a direct relation between early sensory processing and perceived duration, but indicate that subjective duration results from an interaction between objective temporal stimulus features and their conscious perception.
